# Investigation of premenstrual syndrome in connection with physical activity, perceived stress level, and mental status—a cross-sectional study

**DOI:** 10.3389/fpubh.2023.1223787

**Published:** 2023-08-03

**Authors:** Olívia Dózsa-Juhász, Alexandra Makai, Viktória Prémusz, Pongrác Ács, Márta Hock

**Affiliations:** ^1^Faculty of Health Sciences, Institute of Physiotherapy and Sport Sciences, University of Pécs, Pécs, Hungary; ^2^Physical Activity Research Group, Szentagothai Research Centre, University of Pecs, Pécs, Hungary

**Keywords:** premenstrual syndrome, perceived stress, mental health, physical activity, cross sectional research

## Abstract

**Introduction:**

The premenstrual syndrome (PMS) is a critical factor in women’s health, which, in addition to physical inactivity, can be influenced by the body mass index (BMI), stress, and mental state, among others. The study aimed to assess the severity of PMS symptoms among young women regarding physical inactivity, BMI, mental state, and perceived stress level.

**Methods:**

A total of 198 female participants between the ages of 18–45 took part in a 6-month cross-sectional online questionnaire study. The average age of the participants was 25.37 ± 4.80 years. To assess physical activity, stress, mental state, and premenstrual symptoms, we employed standard questionnaires, including the International Physical Activity Questionnaire-Short Form (IPAQ-SF), the Perceived Stress Scale (PSS), the General Health Questionnaire-12 (GHQ-12), and the Premenstrual Assessment Form-Short Form (PAF-SF). The collected data were analyzed using IBM SPSS (Statistical Package for Social Sciences) version 28.0 software, with a significance level set at *p* < 0.05.

**Results:**

During the analysis, we observed a significant relationship (*p* = 0.020) between regular exercise and the severity of PMS symptoms, as well as between mental state and PMS symptoms (*p* < 0.001). Furthermore, our findings revealed a significant negative correlation between regular physical activity and perceived stress levels (r = −0.179; *p* = 0.012), as well as between regular exercise and the participants’ mental state (r = −0.157; *p* = 0.027). Additionally, we identified a significant difference (*p* < 0.001) among the six subgroups formed based on the PAF-SF and average PSS questionnaire results. Moreover, a significant difference was observed between the PAF-SF case and control groups in terms of BMI averages (*p* = 0.019).

**Discussion:**

The research findings indicate that the severity of PMS symptoms is influenced by regular physical activity, mental state, and stress.

## 1. Introduction

The premenstrual syndrome (PMS) significantly impacts women’s health and daily lives (1).

PMS encompasses a diverse array of emotional, behavioral, and physical symptoms that can persist for several days to weeks prior to menstruation and typically subside after the menstrual period commences (1). According to findings from a cohort study conducted in Zurich, approximately 8% of women aged 21–35 experience severe premenstrual symptoms, while an additional 14% experience moderate premenstrual symptoms (2). According to a more recent study among university students published in 2019, 49.9% (*N* = 642) of participants suffered from the severity of premenstrual symptoms (3).

Previous studies have also investigated the nature of PMS, its root causes, comorbidities, and possible remedies. Research findings indicate a correlation between stress and the onset and progression of PMS (4). Electroencephalogram stress tests showed that women with PMS had higher alpha activity and decreased breathing rate when they were under stressful conditions (5). Some studies have observed autonomic nervous system activation in women with PMS by introducing a stressor, for which they used the Montreal Imaging Stress Task, which was able to successfully activate brain areas belonging to the autonomic nervous system (6–8). The results of the study showed that compared to the control group, women with PMS had lower activity in the precentral gyrus and the right middle orbitofrontal gyrus during an acute stress task, while the brain activity in the right middle frontal gyrus was greater during the post-stress recovery process (9). Research was also carried out to find out what hormonal changes are behind certain stress factors. For example, they examined the cortisol awakening response (CAR) in women with PMS and concluded that a low CAR activity profile might be an important factor in the development of PMS (10).

Depression, closely intertwined with stress, represents a significant concern and exerts a substantial influence on the severity of PMS symptoms. A study conducted among university students revealed a high prevalence of PMS, with 58.1% of students experiencing it, particularly among those at risk of depression, where the prevalence was significantly higher (11). A further systematic review has also confirmed that there may be a positive correlation between postpartum depression and premenstrual syndrome, as these two are attributed to a common etiology and pathology (12).

The studies conducted in this area also highlighted a significant correlation between the severity of PMS symptoms and the level of physical activity. The results of a survey showed that the physical and psychological symptoms of the test and control groups indicated a significant difference following a 2-month exercise program. Overall, after 4 weeks of aerobic exercise, PMS was reduced by 31%, physical symptoms by 29%, and psychological symptoms by 33%. After 8 weeks, these reduction rates were 60%, 65%, and 52%, respectively, and these findings indicate that 4 weeks of aerobic exercise can be expected to reduce PMS symptoms. However, the reduction in PMS symptoms after 8 weeks was significantly better than the results observed after 4 weeks (13). The results of another quasi-experimental study conducted in 2013 among women aged 18–32 showed that aerobic exercise and walking effectively reduce the severity of physical symptoms of PMS (14). The symptoms of premenstrual syndrome have already been examined in connection with many factors, but no study has yet been conducted in Hungary that would have examined it in such a complex manner.

Our research aimed to assess the severity of PMS symptoms among young women and investigate how a sedentary lifestyle, body mass index, depression, and stress affect that and how the listed factors influence each other.

## 2. Materials and methods

### 2.1. Participants and procedure

The research was a cross-sectional survey conducted on an electronic interface. The measuring instrument employed consisted of both standard and custom-made questionnaires.

The data collection lasted from September 2022 to March 2023.

The research targeted women aged 18–45 who experienced symptoms of PMS during the parameterization phase. The sampling procedure used was convenience sampling.

Regarding exclusion criteria, the sample could not include women older than 45 years, who are pregnant, who have not menstruated for more than 3 months, or who suffer from premature ovarian failure.

The questionnaire was distributed to a total of 500 individuals, including members of women’s health-focused social media groups and universities in Hungary.

**Figure 1:**
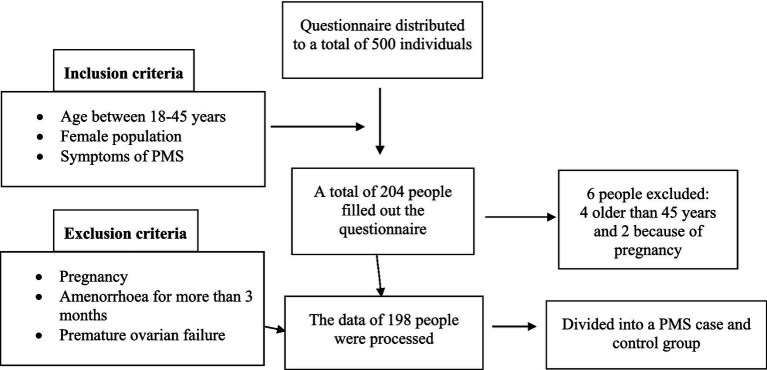
Figure illustrating the sampling process.

A total of 204 people filled out the questionnaire. In accordance with the selection criteria, the data of 198 participants were processed. Based on the exclusion criteria, 6 of them were excluded: 4 because of their age (those older than 45) and another 2 because of pregnancy (Figure 1).

Simultaneously with the cross-sectional study, we established two groups based on the outcomes of the Premenstrual Assessment Form-Short Form questionnaire: a case group and a control group.

### 2.2. Data gathering instruments

The self-administered section of the assessment tool comprised general questions (covering socio-demographic, reproductive, and public health aspects, as well as anthropometric data) and standardized questionnaires embedded within the online survey.

We evaluated the subjects’ age and determined their body mass index (BMI) by measuring their weight and height (15).

During the survey, we examined the subjects’ lifestyle factors, including smoking, coffee, alcohol, and drug consumption habits. We gathered information about the characteristics of their menstrual cycle, such as the presence of pain and cramps, as well as the regularity of the cycle (considering a cycle between 21 and 35 days as regular). Additionally, we assessed the occurrence of pregnancy and breastfeeding.

The survey contained general questions to collect data on the subjects’ physical activity.

An overview of the validated questionnaires used in the research is presented in the following subsections.

#### 2.2.1. The general health questionnaire-12 item version

To assess the presence of depression, we utilized Goldberg’s 12-item General Health Questionnaire (GHQ-12). The original questionnaire, comprising 60 questions, has undergone various useful adaptations over time. The GHQ-12 evaluates mental health across four subscales: somatic symptoms, anxiety and insomnia, social dysfunctions, and severe depression. Participants responded to the 12 questions on a scale ranging from 0 to 3. When evaluating the GHQ-12, two scoring systems are commonly employed: the bimodal (0-0-1-1) and the Likert scoring (0-1-2-3). The cut-off point for the bimodal scoring method is 2/3, with a maximum score of 12, while for the Likert scale, it is 8/9, with a maximum score of 36 (16, 17).

#### 2.2.2. The perceived stress scale

To evaluate perceived stress, we employed the validated Hungarian-language version of the Perceived Stress Scale (PSS). This questionnaire provides insights into the level of stress experienced in the past month. It consists of 10 questions, which respondents rate on a 5-point scale ranging from 0 to 4. Therefore, the lowest possible score is 0, and the highest is 40. During the assessment, based on the total scores, respondents can be categorized into three groups: 0–13 points indicate a low level of perceived stress, 14–26 points show moderate perceived stress and 27–40 points reveal a high level of perceived stress (18, 19).

#### 2.2.3. The international physical activity questionnaire-short form

We used the shortened version of the International Physical Activity Questionnaire (IPAQ-SF) to assess physical activity, validated in Hungarian. The questionnaire assesses the number of days and hours the subjects spend in heavy, moderate, or light physical work and examines the time all activities spent walking, lying down, and sitting for a period of 1 week (20).

#### 2.2.4. The premenstrual assessment form—short form

We utilized the Hungarian-translated version of the shortened Premenstrual Assessment Form (PAF-SF), which comprises 10 questions, to assess premenstrual symptoms. The questionnaire includes three subscales. The first subscale, “Affect,” examines increased irritability, bad mood, sad mood, stress, and feeling overworked. The second subscale focuses on “Water retention,” addressing issues like edema, limb swelling, bloating, and weight gain. The third and final subscale assesses “Pain,” encompassing lower abdominal cramps, back pain, joint pain, and breast tenderness. It is important to note that all questions pertain to the 7–14 days preceding menstruation, and respondents rate their experiences on a scale of one to six. The scale values are as follows: 1 = uncharacteristic or no change, 2 = minimal change, 3 = slight change, 4 = moderate change, 5 = severe change, 6 = very severe change. The total score is calculated based on the responses to these questions, ranging from a minimum of 10 to a maximum of 60. According to several international studies, the diagnostic threshold is set at 27, and this criterion was employed to evaluate the gathered data (21).

Following the analysis of the PAF questionnaire results, we formed a control group and a case group to investigate individual factors associated with PMS symptoms.

### 2.3. Data analysis

For descriptive statistics, we performed various statistical calculations, including minimum, maximum, mean (± standard deviation), and median (interquartile range) to provide characterization. The normality test was conducted using the Kolmogorov–Smirnov test. Based on the results, for examining the relationships between the factors, we employed Spearman correlation analysis and Pearson chi-square test to determine the impact of the influencing factors (PSS, GHQ-12, IPAQ-SF) of PMS multivariate linear regression analysis adjusted for age and BMI was applied furthermore, independent sample *T*-test and Mann–Whitney *U*-test was applied to examine and evaluate the differences between the case and control groups and subgroups of PMS.

The statistical analysis of the study involved the use of Microsoft Excel 2016 and IBM SPSS version 28.0 (SPSS Inc., Chicago, IL, United States) as software tools.

The significance level was set at *p* < 0.05.

### 2.4. Ethical approval and consent to participate

The study was approved by the Institutional Review Board of the Regional Research Committee of the Clinical Center, Pécs, Hungary (No.: 9386-PTE 2022). Clinical Trials ID: NCT05811130. All the methods used were carried out under relevant guidelines and regulations. The data were processed anonymously and confidentially based on the Data Protection Act of Hungary.

The research was conducted in accordance with the principles of the Declaration of Helsinki: the subjects voluntarily agreed to the study, the participants were fully informed before the data collection phase, and they provided a written Declaration of Consent.

## 3. Discussion

The results of our study conducted on the average population showed that 53% of the participants suffered from moderate or severe PMS symptoms. Regarding the prevalence of PMS, according to a 2001 study, 14% of women suffer from PMS (2), while as stated in a recent student survey in 2019, nearly 50% of participants suffer from PMS, which causes severe changes in the quality of life (3).

Our research findings revealed a notable distinction in the body mass index (BMI) averages between the PAF case and control groups, despite both groups falling within the normal range. A study conducted in 2015 recognized the association of various factors with premenstrual symptoms (PMS), encompassing both physical and psychological aspects. The study aimed to explore the relationship between cardiorespiratory endurance (CRF), regular physical activity (including exercise and sedentary behavior), BMI, and the physical and psychological symptoms of PMS. The results of their investigation led them to conclude that higher levels of CRF and engagement in physical activity were linked to reduced PMS symptoms, whereas higher BMI values were associated with more pronounced PMS symptoms (22). These findings can be compared with our research results.

Lifestyle factors can greatly influence the severity of PMS symptoms. The results of a current study showed a strong relationship between PMS and fried foods (*p* = 0.017), sweet drinks (*p* = 0.018), fast food (*p* = 0.048), fruit (*p* = 0.012), no habitual exercise (*p* = 0.006), family history of PMS (*p* = 0.002), hip circumference (*p* = 0.04), and body mass index (*p* = 0.04).

Our results confirm the assumption that regular physical activity has a beneficial effect on the symptoms of premenstrual syndrome (*p* = 0.02). During previous research, the extent to which 3 months of aerobic and walking-based physical exercises influence the physical and mental symptoms of PMS and pain caused by menstruation was examined. The results revealed a significant difference in physical symptoms after one menstrual cycle (14). Furthermore, research examining the impact of aerobic exercise also showed that even 4 weeks of physical activity could have a beneficial effect on reducing PMS symptoms. However, the best results can be expected from aerobic physical activity performed for at least 8 or more weeks (13).

Our study also indicated a significant relationship between the tendency to depression and the strength of PMS symptoms (R = 0.485; *p* < 0.001). The aim of a 2016 study conducted on 618 university students was to determine the frequency and influencing factors of premenstrual syndrome in first-year health students and examine the significance of the relationship between depression and PMS. The results included that the prevalence of PMS was significantly higher among students at risk of depression (11).

Examining the connection between PMS and perceived stress, the Spearman correlation analysis showed a significant positive relationship (R = 0.395; *p* < 0.001). A similar study of 215 college students aimed to identify associations between perceived stress, psychological resilience, and PMS and examine factors influencing PMS. Among their results obtained with Pearson correlation and regression analysis, it should be highlighted that PMS was positively correlated with perceived stress (23). The mentioned research results can be paralleled with those presented in our research.

Our research found a significant negative correlation between depression and physical activity (R = −0.157; *p* = 0.027); this can be paralleled with a previous meta-analysis, as a result of which a recent overview of the beneficial effect of exercise as an effective tool for the prevention and treatment of depression was given (24).

We discovered a significant negative correlation between regular physical activity and perceived stress level (R = −0.197; *p* = 0.012), which is also confirmed by the results found in the international literature (25). The outcome of their research—as well as our results—suggest that there is increasing evidence that stress can negatively influence the willingness to engage in physical activity.

Based on the results of the multivariable linear regression model, the amount of perceived stress had a significant impact on premenstrual symptoms, while the effect of physical activity on premenstrual symptoms was confirmed indirectly through the correlation of perceived stress level.

Judging from the test results, during the third wave of COVID-19, the total activity of the women participating in this research was, on average, 695.88 min/week (*N* = 198). Our study was supported by a domestic research result, where the total physical activity of the participants dropped to 686 min/week during the third wave (26).

This preceding research also assessed the perceived stress level among the Hungarian population with the PSS questionnaire. The data measured during this research were also supported by other Hungarian research, as the average value we determined was 19.61 points (*N* = 198). The standard value of perceived stress was 26.40 points, compared to the one measured during the third wave of the Hungarian research, which was 20.09 points (18, 26).

Our research found a significant positive correlation between the perceived stress level and the tendency to depression (R = 0.803; *p* < 0.001). This result of our study is supported by many research findings presented in international publications (27, 28).

## 4. Results

The average age of the examined persons was 25.37 ± 4.80 years, and their average body height was 167.92 ± 5.71 cm. Their average body weight was 63.16 ± 13.42 kg, and the average body mass index was 22.38 ± 4.63 kg/m^2^.

The following table shows the main characteristics of the people who participated in the research, important from the point of view of the investigation, in percentage distribution (Table 1).

**Table 1:** Frequency of the main characteristics of the examined group in percentage distribution (*N* = 198).

Characteristics	Percentage distribution (*N* = 198)
Smoking	11.1% (*N* = 22)
Coffee consumption	65.2% (*N* = 129)
Has irregular menstruation	21.2% (*N* = 42)
On a scale from 1 to 5, the severity of the bleeding is rated as 3	46.5% (*N* = 92)
The most common answer to the length of menstrual bleeding: are 5 days	35.9% (*N* = 71)
Has intermittent bleeding	11.1% (*N* = 22)
Has painful and spasmodic menstruation	81.3% (*N* = 161)
Reported a pain of at least 7 on the VAS scale during the bleeding	19.7% (*N* = 39)
Taking birth control pills	26.3% (*N* = 52)
Has a previous diagnosis of PCOS	14.6% (*N* = 29)
Gave birth at least once	12.6% (*N* = 25)
Had an artificial or spontaneous abortion	4.0% (*N* = 8)
Has a previous diagnosis of depression	5% (*N* = 10)
Had surgical intervention	39.4% (*N* = 78)
The most common surgical intervention was: tonsillectomy (*n* = 27); other: knee surgery (*n* = 6)	
Uses a relaxation method	16.6% (*N* = 33)
Most common: meditation (*n* = 15); other: yoga (*n* = 9)	
Uses a sedative	4.5% (*N* = 9)
Most common: valerian (*n* = 4); other: Xanax, Frontin, lemongrass tea (*n* = 5)	

### 4.1. Presentation of the evaluation and results of standardized tests on the average Hungarian population

Based on the results of PAF-SF, 53% (*N* = 105) of the subjects were positive, and 47% (*N* = 93) were classified as negative for PMS.

As a consequence of the evaluation of the GHQ-12, 60.1% (*N* = 119) of the subjects were considered positive regarding their mental state, and 39.9% (*N* = 79) were regarded as negative.

The obtained results of the IPAQ-SF showed that 41.4% (*N* = 82) of the respondents had performed intense exercise, and only 9.6% (*N* = 19) had performed low-intensity exercise in the past week, another 49% (*N* = 97) fell into the category of moderate physical activity.

Based on the total scores of PSS, using the grouping according to the given point limits, we found that 55.6% (*N* = 110) of the subjects suffer from moderate levels of stress. In comparison, 19.7% (*N* = 39) suffer from a high level of perceived stress in everyday life, and another 24.7% (*N* = 49) were classified as having low-level perceived stress.

### 4.2. Presentation of the correlations between the results of the individual standardized questionnaires

The tables below show the average and standard deviation of the results of the standard questionnaires and other measuring instruments used, as well as the relationships between them (Tables 2 3).

**Table 2:** Presentation of the arithmetic characteristics of the results of the standard questionnaires and other measuring instruments used (*N* = 198).

	Mean	Standard deviation	Minimum, maximum	Median	Interquartile range
BMI (kg/m^2^)	22.38	4.63	16.22; 44.98	21.15	19.46; 24.16
GHQ-12 (points)	4.43	3.58	1.00; 8.00	4.00	1.00; 8.00
PAF-SF (points)	28.08	9.49	21.00; 35.00	27.00	21.00; 35.00
PSS (points)	19.61	7.98	13.75; 26.00	20.00	13.75; 26.00
IPAQ-SF (min/week)	695.88	732.96	277.50; 791.25	510.00	277.50; 791.25

**Table 3:** Presentation of the relationships between the results of the standard questionnaires and other measuring instruments (*N* = 198).

Variable	Test value and singnificance
IPAQ-SF and PSS	R = −0.197; *p* = 0.012^*^
IPAQ-SF and GHQ-12	R = −0.157; *p* = 0.027^*^
PSS and GHQ-12	R = 0.803; *p* < 0.001^*^
PSS and PAF-SF	R = 0.395; *p* < 0.001^*^
GHQ-12 and PAF-SF	R = 0.485; *p* < 0.001^*^
IPAQ-SF and BMI	*χ*² = 27.701; *p* = 0.001^**^
IPAQ-SF and PAF-SF	*χ*² = 5.584; *p* = 0.02^**^
PAF-SF and GHQ-12	*χ*² = 27.075; *p* < 0.001^**^
PAF-SF and menstrual cramps	*χ*² = 9.918; *p* = 0.002^**^
PAF-SF 6 subcategories and PSS	T = −5.019; *p* < 0.001^***^
PAF-SF case and control group and BMI	Z = −2.346; *p* = 0.019^****^

^*^Spearman correlation analysis; ^**^Pearson’s Chi-square test; ^***^Independent sample *T*-test; ^****^Mann–Whitney *U*-test.

We built a linear regression model to investigate the relationships between the examined factors, such as stress, premenstrual symptoms and physical activity. Based on our results, the level of perceived stress had a significant effect on premenstrual symptoms (R^2^ = 0.154; *F* = 9.963, *p* < 0.001; B = 0.478, *p* < 0.001). We were able to verify the effect of physical activity on perceived stress with a multivariate model (R^2^ = 0.579; *F* = 68.853, *p* < 0.001; B < 0.001, *p* = 0.010).

### 4.3. Comparison of the results of PMS case and control groups based on the premenstrual assessment form short form questionnaire in Hungary

A notable disparity was observed in the average body mass index (BMI) between the two groups (*p* = 0.019). The control group had a mean BMI of 21.78 ± 4.40 kg/m^2^, whereas the case group had a mean BMI of 22.91 ± 4.77 kg/m^2^.

Moreover, a significant association was observed between the PMS case and control groups and the variables of the 12-item General Health Questionnaire (GHQ-12; *p* < 0.001). Within the PMS control group, 59.1% of the participants exhibited no inclination toward depression, whereas 77.1% of the subjects in the PMS case group were identified as being susceptible to depression.

It was also shown that there is a significant relationship between painful cramps accompanying menstruation and PMS symptoms (*p* = 0.002). In the PMS control group, 72.0% of the subjects stated that they have painful cramps during menstruation, while 89.5% of the participants in the PMS case group answered yes to the same question.

It has been proven that the average scores of the Perceived Stress Questionnaire differ significantly (*p* < 0.001) in each PAF subcategory (6 categories created by the authors). The PSS scores of the PAF subcategories are as follows in Table 4.

**Table 4:** Presentation of the PSS scores of the PAF subcategories (*N* = 198).

PAF-SF subcategories	Mean and standard deviation (points)	Distribution (*N*; %)
1.	9.00 ± 0.00	1; 0.50
2.	16.50 ± 8.77	44; 22.22
3.	17.92 ± 7.15	78; 39.39
4.	21.80 ± 6.64	51; 25.75
5.	26.18 ± 6.46	22; 11.11
6.	30.50 ± 0.70	2; 1.01

### 4.4. Strengths and limitations

The strengths of the research include that there has not been a similarly comprehensive study on the topic regarding the Hungarian population before this study.

It should be emphasized that the IPAQ-SF assesses all physical activity and does not separately ask about leisure activities or physical activity for recreational purposes.

This research also had limitations. The willingness to fill in online questionnaire research is low, and the size of the sample is not representative enough; ergo, it is necessary to carry out similar studies on a larger sample in the future.

### 4.5. Conclusion

Based on our findings, we observed a significant positive correlation between PMS and depression, stress and depression, and stress and PMS symptoms. Furthermore, a significant negative correlation was detected between the level of physical activity and the propensity for depression, as well as between the level of physical activity and perceived stress levels.

Additionally, our subsequent findings revealed a significant correlation between the subcategories of BMI and levels of physical activity. Furthermore, we identified a significant relationship between the severity of premenstrual syndrome symptoms and the levels of physical activity.

The findings revealed a significant disparity in the body mass index (BMI) averages between the PAF case and control groups. Additionally, the Pearson chi-square test indicated a significant relationship between the PMS case and control groups concerning the average scores of the GHQ-12. Furthermore, a significant connection was observed between the presence of painful cramps during menstruation and PMS symptoms.

In summary, we can conclude that physical activity level, body mass index, the tendency to depression, and perceived stress level are in relationship with the severity of PMS symptoms, and physical activity is related to changes in body mass index.

## Data availability statement

The raw data supporting the conclusions of this article will be made available by the authors, without undue reservation.

## Ethics statement

The studies involving human participants were reviewed and approved by Institutional Review Board of the Regional Research Committee of the Clinical Center, Pécs, Hungary. The patients/participants provided their written informed consent to participate in this study.

## Author contributions

OD-J, AM, MH, PÁ, and VP contributed to the conception and design of the study. OD-J, AM, and MH wrote sections of the manuscript. All authors contributed to the article and approved the submitted version.

## Funding

This project was supported the ÚNKP-22-4-II-PTE-1681 New National Excellence Program of the Ministry for Culture and Innovation from the source of the National Research, Development and Innovation Fund, and TKP-2021-EGA-10 project of the National Research, Development and Innovation Fund of Hungary, financed under the TKP-2021-EGA funding scheme.

## Conflict of interest

The authors declare that the research was conducted in the absence of any commercial or financial relationships that could be construed as a potential conflict of interest.

## Publisher’s note

All claims expressed in this article are solely those of the authors and do not necessarily represent those of their affiliated organizations, or those of the publisher, the editors and the reviewers. Any product that may be evaluated in this article, or claim that may be made by its manufacturer, is not guaranteed or endorsed by the publisher.
